# Anti-inflammatory Activity and PGE_2_ Inhibitory Properties of Novel Phenylcarbamoylmethyl Ester-Containing Compounds

**DOI:** 10.3390/molecules14020667

**Published:** 2009-02-11

**Authors:** Flora Barsoum, Hanan Georgey, Nagwa Abdel-Gawad

**Affiliations:** Pharmaceutical Chemistry Department, Faculty of Pharmacy, Cairo University, Kasr El-Eini Street, Cairo 11562, Egypt; E-mails: ffbarsoum2@yahoo.com (F.B.), hamadido21@hotmail.com (N.A-G.)

**Keywords:** Ibuprofen, Naproxen, 4-(Un)substituted phenylcarbamoylmethyl esters, Anti-inflammatory, Ulcerogenic liability, PGE_2_

## Abstract

A variety of 4-(un)substituted phenylcarbamoyl methyl ester-containing compounds **3a-d**, **5a-d** and **7a-d** were synthesized via reaction in *N*,*N*-dimethylformamide of (un)substituted chloroacetanilides **2a-d** with the potassium salts of ibuprofen (**1**), naproxen (**4**) and *N*-acetylanthranilic acid (**6**). Moreover, other 4-(un)substituted phenylcarbamoylmethyl ester-containing compounds **10a-d** were synthesized via the attack of (un)substituted chloroacetanilides **2a-d** on one of the carboxylic acid groups of the potassium salt of 4-(2-carboxyethylcarboxamido)benzoic acid (**8**) in *N*,*N*-dimethylformamide, with subsequent cyclization of the other one giving finally a pyrrolidinone structure. Anti-inflammatory properties of the synthesized compounds were evaluated in *vivo* utilizing a standard acute carrageenan-induced paw oedema method in rats and the most promising prepared anti-inflammatory active agents were evaluated for ulcerogenic liability in rats using ibuprofen and naproxen as reference standards in both screenings. PGE_2_ inhibitory properties of the highly promising anti-inflammatory agents synthesized and low gastric ulcerogenic liabilities were tested with a PGE_2_ assay kit technique.

## Introduction

Non-steroidal anti-inflammatory drugs (NSAIDs) are widely used in the treatment of pain and inflammation. Most currently used NSAIDs suffer from limitations in their therapeutic uses, since they cause gastrointestinal and renal side effects, which are inseparable from their pharmacological activities. These compounds act via inhibition of the enzyme cyclooxygenase, thus preventing prostaglandin synthesis [1]. They have greater selectivity to inhibit COX-1 (constitutively expressed and providing cytoprotection in the gastrointestinal tract and necessary for normal platelet aggregation and renal function) than COX-2 (inducible by inflammatory stimuli) [2,3,4,5]. Consequently, long-term therapy with non-selective NSAIDs may cause gastrointestinal complications ranging from stomach irritation to life-threatening gastrointestinal ulceration and bleeding [6]. Several lines of evidence have suggested that modification of the carboxyl function of representative NSAIDs results in retained anti-inflammatory activity and reduced ulcerogenic potential [7,8,9].

In the present work, we intended to investigate the synthesis of 4-(un)substituted phenylcarbamoylmethyl ester-containing compounds adopting simple synthetic approaches and utilizing easily accessible starting chemicals. These compounds are esters of well known NSAIDs (ibuprofen and naproxen), *N*-acetylanthranilic acid (nitrogen isostere of salicyclic acid [10]) and 4-(2,5-dioxopyrrolidin-1-yl)benzoic acid, in which the carboxylic function is altered to a substituted ester prodrug residue hoping to circumvent the untoward ulcerogenic side effects of the free acid. Also, (un)substituted phenylcarbamoylmethyl moieties were made to study the effect of such substituents on the physicochemical properties of the compounds and consequently on their pharmacological activity, to produce new hit compounds for a drug discovery program. The anti-inflammatory properties of the synthesized compounds were screened. Ulcerogenic liabilities for the most active anti-inflammatory compounds in each group would be considered. Additionally, PGE_2_ inhibitory properties for the most active anti-inflammatory agents with less ulcerogenic liability in each group were determined since PGE_2_ stimulates tumor cell proliferation and differentiaton [11,12] and cancer regression has been documented in patients taking non-steroidal, anti-prostaglandin drugs such as ibuprofen [13]. The interest for designing these compounds is attributed to the biological and pharmacological properties associated with their structures. In addition to their anti-inflammatory activities [14,15], ibuprofen affects the amyloid pathology in the brain [16] and its long-term use has been associated with reduced risk of neurodegeneration [17]. Moreover, a variety of pyrrolidinone derivatives were reported to possess antibacterial [18], anticonvulsant [19] and anticancer [20] activities.

## Results and Discussion

### Chemistry

**Scheme 1:**
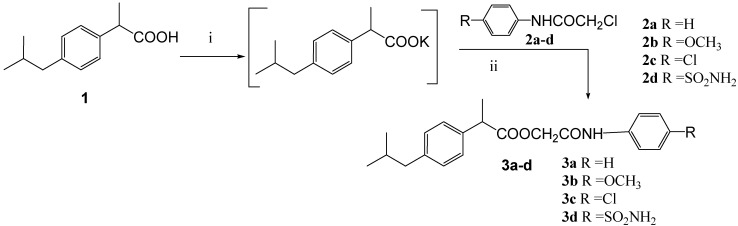
Synthetic Pathway for Compounds **3a-d**.

**Scheme 2:**
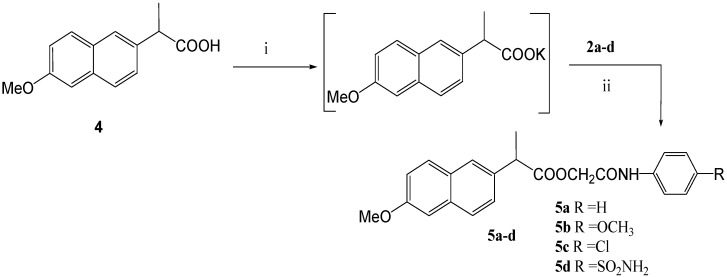
Synthetic Pathway for Compounds **5a-d**.

**Scheme 3:**
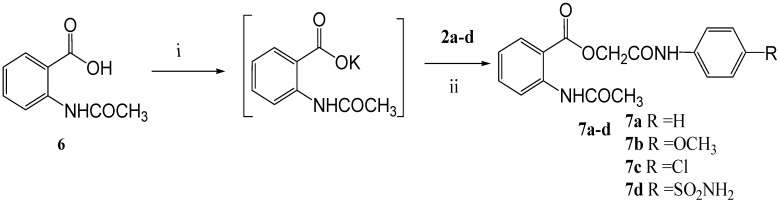
Synthetic Pathway for Compound **7a-d**.

Reaction of the potassium salts of ibuprofen (**1**), naproxen (**4**) and *N*-acetylanthranilic acid (**6**) with (un)substituted chloroacetanilides **2a-d** in *N*,*N*-dimethylformamide (Scheme 1, Scheme 2, and Scheme 3) afforded the corresponding 4-(un)substituted phenylcarbamoylmethyl esters **3a-d**, **5a-d**, and **7a-d** in good yields (80-89%). 

*Reaction conditions*: i) KOH, ethanol, ii) DMF

*Reaction conditions*: i) KOH, ethanol, ii) DMF

*Reaction conditions*: i) KOH, ethanol, ii) DMF

The structures of **3a-d**, **5a-d** and **7a-d** were established through spectroscopic (IR, ^1^H-NMR, MS) as well as elemental analyses data. The IR spectra of **3a-d**, **5a-d** and **7a-d** revealed the presence of a strong band (at *ν* = 1758-1740 cm^-1^ for **3a-d**, at *ν* = 1749-1736 cm^-1^ for **5a-d** and at *ν* = 1709-1658 cm^-1^ for **7a-d**, respectively), assignable to their carbonyl ester functions, in addition to the NH amidic stretching vibration band (at *ν* = 3400-3206 cm^-1^ for **3a-d**, at *ν* = 3389-3249 cm^-1^ for **5a-d** and at *ν* = 3424-3285 cm^-1^ for **7a-d**, respectively).

The ^1^H-NMR spectra of **3a-d** and **5a-d** reveal the presence of the methylene group as two doublet signals (at *δ* = 4.47-4.52, 4.90-4.93, *J* = 15.3-15.9 for **3a-d** and at *δ* = 4.44-4.47, 4.92-5.03, *J* = 15.3-15.9 for **5a-d**, respectively), due to their mutual coupling with each other since there is a chiral center and the two protons are non-equivalent. In contrast, in the case of **7a-d** this methylene residue appears as a sharp singlet signal at *δ* = 4.94-5.00 due to the absence of chirality. The mass spectra of **3a-d**, **5a-d** and **7a-c** exhibited the expected parent molecular ion peaks, thus confirming the assumed structures.

**Scheme 4:**
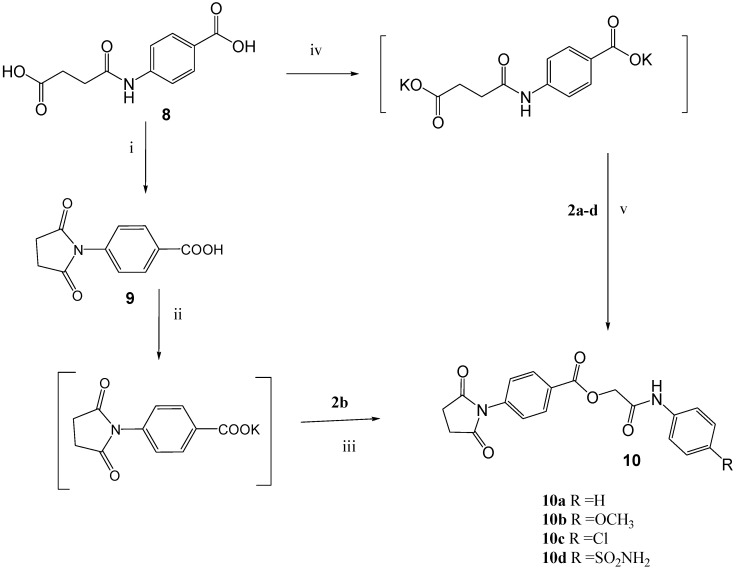
Synthetic Pathway for Compound **10a-d**.

Meanwhile, 4-(un)substituted phenylcarbamoylmethyl esters **10a-d** were obtained through reaction of (un)substituted chloroacetanilides **2a-d** with the potassium salt of 4-(2-carboxyethyl-carboxamido)benzoic acid **8** in *N*,*N*-dimethylformamide. The reaction was assumed to take place at both carboxylic acid groups giving two phenylcarbamoylmethyl ester moieties. However, different spectroscopic data reveal that the chloroacetanilides attacked only one of the carboxylic acid groups, with subsequent cyclization of the other one finally gave the pyrrolidinone structure in spite of the use of 2 moles KOH and 2 moles of (un)substituted chloroacetanilides **2a-d** per mole of the starting material **8** (Scheme 4).

*Reaction conditions*: i) (CH_3_CO)_2_O ii) KOH, DMF, iii) DMF, iv) KOH, ethanol, v) DMF

The structures of **10a-d** were confirmed through spectroscopic (IR, ^1^H-NMR, MS) as well as elemental analyses data. The IR spectra of **10a-d** exhibit strong carbonyl bands at *ν* = 1778-1782, 1706-1719, 1605-1690 cm-1, corresponding to the pyrrolidinone (cyclic amide), ester and acyclic amide moieties, respectively. The ^1^H-NMR spectra of **10a-d** strongly support the presumed cyclized structures, exhibiting only one D_2_O exchangeable amino signal at *δ* = 10.05-10.56, a sharp singlet signal at *δ* = 2.81-2.82 corresponding to the two pyrrolidinyl methylene protons besides a singlet signal of one methylene ester function at *δ* = 4.91-4.99. The ^13^C-NMR (APT) spectrum of **10b** excludes any other possible structure, revealing the presence of carbonyl groups corresponding to two cyclic amides at *δ* = 177.19, one ester at *δ* = 165.46 and an acyclic amide at *δ* = 165.54, confirming the cyclization reaction process. The mass spectra of **10a-d** exhibiting the parent molecular ion peaks confirmed the assumed structures. To further confirm the cyclization reaction, a chloroacetanilide, i.e. **2b**, was reacted with the potassium salt of 4-(2,5-dioxopyrrolidin-1-yl)benzoic acid (**9**) in *N*,*N*-dimethylformamide to give the same compound **10b**.

### Anti-inflammatory Activity

The anti-inflammatory activity of all synthesized compounds **3a-d**, **5a-d**, **7a-d** and **10a-d** was determined by the standard carrageenan-induced paw oedema method in rats [21,22,23], at a dose of 50 mg/kg body weight, and compared with ibuprofen and naproxen as reference drugs. From the results obtained (Table 1), it appears that all ibuprofen containing compounds **3a-d** show slightly enhanced anti-inflammatory activities (78.2-89.5% inhibition of oedema) comparable to that of the reference drug ibuprofen (76.1%). Similarly, all naproxen containing compounds **5a-d** also exhibit better activities (83.6-88.3%) relative to the reference drug naproxen (75.3%). On the contrary, all *N*-acetyl anthranilic acid derivatives **7a-d** possess anti-inflammatory activities (54.2-67.3%) lower than both ibuprofen and naproxen. Moreover, among compounds having the pyrrolidinone ring, **10b** and **10d** exhibit better anti-inflammatory activity (84.1% and 79.4%, respectively) than both ibuprofen (76.1%) and naproxen (75.3%).

**Table 1:** Anti-inflammatory Activity of the Tested Compounds Using Acute Carrageenan-Induced Paw Oedema in Rats.

Compound	Mean swelling volume "mL"	% Inhibition of oedema
**Control**	0.742 ± 0.096^b,c^	00.0
**Ibuprofen**	0.177 ± 0.065^a^	76.1
**Naproxen**	0.183 ± 0.023^a^	75.3
**3a**	0.162 ± 0.103^a^	78.2
**3b**	0.078 ± 0.073^a,b,c^	89.5
**3c**	0.090 ± 0.068^a^	87.9
**3d**	0.132 ± 0.055^a^	82.2
**5a**	0.087 ± 0.031^a,c^	88.3
**5b**	0.113 ± 0.043^a^	84.8
**5c**	0.090 ± 0.058^a^	87.9
**5d**	0.122 ± 0.074^a^	83.6
**7a**	0.243 ± 0.101^a^	67.3
**7b**	0.245 ± 0.107^a^	67.0
**7c**	0.320 ± 0.078^a,b,c^	56.9
**7d**	0.340 ± 0.105^a,b,c^	54.2
**10a**	0.208 ± 0.080^a^	72.0
**10b**	0.118 ± 0.085^a^	84.1
**10c**	0.338 ± 0.123^a,b,c^	54.4
**10d**	0.153 ± 0.119^a^	79.4

^a^ Significantly different from the control value at *p* < 0.05; ^b^ significantly different from the ibuprofen value at *p* < 0.05; ^c^ significantly different from the naproxen value at *p* < 0.05; results are means of six experiments ± SE.

### Ulcerogenic Liability

The ulcerogenic liability for the most active anti-inflammatory compounds (**3b**, **3c**, **5a**, **5c**, **7a** and **10 b**) in each series was determined in albino rats following the previously reported method [23,24]. From the data obtained (Table 2), it has been observed that all the tested compounds possess less ulcerogenic potentialities (ulcer indexes of 11.60-16.69), compared with that of the standard drugs ibuprofen and naproxen (ulcer indexes of 22.90 and 23.15, respectively).

**Table 2:** Ulcergenic Liability of Selected Compounds.

Compound	Number of animals with ulcers	% incidence divided by 10	Average number of ulcers	Average severity	Ulcer index
**Control**	0/5	0.00	0.00	0.00	0.00
**Ibuprofen**	5/5	10	11.00	1.90	22.90
**Naproxen**	5/5	10	11.40	1.75	23.15
**3b**	5/5	10	2.00	1.10	13.10
**3c**	4/5	8	2.60	1.00	11.60
**5a**	4/5	8	3.20	1.00	12.20
**5c**	5/5	10	5.40	1.29	16.69
**7a**	5/5	10	3.40	1.00	14.40
**10b**	4/5	8	5.60	1.70	15.30

### PGE_2_ Inhibitory Properties

PGE_2_ inhibitory properties were studied for compounds **3c**, **5a**, **7a** and **10b**, which showed the highest anti-inflammatory and lowest ulcerogenic properties in each series. This was evaluated *in vivo*, utilizing the previously reported six-day-old air pouch method [25,26]. PGE_2_ was measured using ELISA kit specific for rats, provided by R&D Systems (Minneapolis, USA & Canada), and the results were compared with indomethacin chosen as a reference drug according to the instructions of the kit. From the obtained results (Table 3), it has been observed that all the tested compounds reduce PGE_2_ levels, however, the ibuprofen and naproxen containing compounds (**3c** and **5a**) showed remarkable inhibitions (50.83, 63.83 pg/mL, respectively) compared to indomethacin (98.33 pg/mL). Although compounds **7a** and **10b** exhibited inhibitions of 127.33 and 103.50 pg/mL, respectively, which is lower than indomethacin (98.33 pg/mL), it is remarkably high compared to the control (533.33 pg/mL), indicating the anti-inflammatory activities of these two compounds.

**Table 3:** PGE_2_ Concentration of Selected Compounds.

Compound	Mean concentration "pg/ml"
**Control**	533.33±19.26
**Indomethacin**	98.33±3.33*
**3c**	50.83±3.86*
**5a**	63.83±3.87*
**7a**	127.33±2.53*
**10b**	103.50±2.29*

*Significantly different from the control value at *p* < 0.05. Results are means of 6 experiments ± SE.

## Conclusions

The synthesis of 4-(un)substituted phenylcarbamoylmethyl ester-containing compounds **3a-d**, **5a-d**, **7a-d** and **10a-d** was undertaken. The structure of the newly synthesized compounds was established by microanalytical and spectral (IR, ^1^H-NMR, mass) data. They were tested for their anti-inflammatory activity. Further the ulcerogenic liability and PGE_2_ inhibitory properties for the most active compounds were determined. Results showed that all the tested compounds exhibited promising anti-inflammatory activity, compared to ibuprofen and naproxen, with marked decreases in the ulcerogenic side effects. Moreover, esterification of both ibuprofen and naproxen derivatives led to increases in the anti-inflammatory activity, compared to the parent drugs, and this was enhanced in the case of the 4-methoxyphenylcarbamoyl methyl ester **3b** and the phenylcarbamoylmethyl ester **5a** of ibuprofen and naproxen, respectively. On the other hand, there is a significant change in the pharmacological activity amongst the different ester substituted derivatives of *N*-acetylanthranilic acid **7a-d** and 4-(3-carboxypropionylamino)benzoic acid **10a-d** in which the 4-chloro derivatives **7c** and **10c**, together with the 4-sulphamoyl derivative **7d** showed a marked decrease in the anti-inflammatory activity. Finally, the results of the *in vivo* anti-inflammatory activity by the standard acute carrageenan-induced paw oedema method in rats revealed remarkable activities that largely coincide with the results observed using a PGE_2_ assay kit technique. 

## Experimental

### General

Melting points are uncorrected and recorded on a Gallenkamp melting point apparatus. IR spectra (KBr) were recorded on a Bruker Vector 22 spectrophotometer. ^1^H-NMR spectra were recorded on a Varian MERCURY 300 (300 MHz) spectrometer. Mass spectra were recorded on GCMS-QP 1000 EX, Gas chromatograph-Mass spectrometer, at 70 eV. Compounds **2a-d** [27,28], **6** [29], **8** [30] and **9** [31] were prepared according to the previously reported procedures. Ibuprofen and naproxen were purchased from Sigma Chemical Co. (St. Louis, MO, USA) in the form of racemic mixtures.

### Synthesis of 4-(un)substituted phenylcarbamoylmethyl esters ***3a-d***, ***5a-d*** and ***7a-d***

A mixture of equimolar amounts of **1**, **4**, or **6** and an ethanolic solution of potassium hydroxide (5 mmol) in absolute ethanol (30 mL) was stirred at room temperature (25-30 °C) for 15 minutes. The residue after evaporation under reduced pressure was treated with the appropriate chloroacetanilides **2a-d** (5 mmol) in *N*,*N*-dimethylformamide (5 mL) in a boiling water bath for 12 hours. The reaction mixture was then cooled, poured onto ice-cooled water and the separated solid was collected, dried and crystallized from a suitable solvent affording **3a-d**, **5a-d** and **7a-d** as colourless crystals.

*4-(2-Methylpropyl)-α-methylbenzene acetic acid phenylcarbamoylmethyl ester* (**3a**): m.p. 78-80 °C (from methanol); yield 83%; IR *ν*_max_/cm^-1^: 3330 (NH), 2950, 2922, 2866 (CH aliph.), 1740 (C=O ester), 1685 (C=O amide), 1603, 1546 (C=C); ^1^H-NMR *δ*/ppm (CDCl_3_): 0.92 (d, 6H, 2CH_3_, *J* = 6.6 Hz), 1.59 (d, 3H, CH-*CH_3_*, *J* = 7.2 Hz), 1.85-1.90 (m, 1H, *CH*-CH_2_), 2.49 (d, 2H, CH-*CH_2_*, *J* = 7.2 Hz), 3.85 (q, 1H, *CH*-CH_3_, *J* = 7.2 Hz), 4.49 (d, 1H, upfield H of COO*CH_2_*, *J* = 15.6 Hz), 4.92 (d, 1H, downfield H of COO*CH_2_*, *J* = 15.9 Hz), 7.07-7.30 (m, 10H, arom. H + NH); MS: *m/z* (%) = 339 (M^+^, 20), 247 (6), 188 (96), 161 (100), 93 (37); Anal. Calcd. For C_21_H_25_NO_3_ (339.42): C, 74.31; H, 7.42; N, 4.13%. Found: C, 74.17; H, 7.63; N, 3.94%.

*4-(2-Methylpropyl)-α-methylbenzene acetic acid (4-methoxyphenylcarbamoyl)methyl ester* (**3b**): m.p. 122-124 °C (from 2:1 v/v ether-petroleum ether); yield 87%; IR *ν*_max_/cm^-1^: 3400 (NH), 2960, 2869, 2842 (CH aliph.), 1746 (C=O ester), 1694 (C=O amide), 1600, 1538 (C=C); ^1^H-NMR *δ*/ppm (CDCl_3_): 0.90 (d, 6H, 2CH_3_, *J* = 6.6 Hz), 1.58 (d, 3H, CH-*CH_3_*, *J* = 7.2 Hz), 1.83-1.88 (m, 1H, *CH*-CH_2_), 2.48 (d, 2H, CH-*CH_2_*, *J* = 7.2 Hz), 3.79 (s, 3H, O*CH_3_*), 3.84 (q, 1H, *CH*-CH_3_, *J* = 7.2 Hz), 4.48 (d, 1H, upfield H of COO*CH_2_*, *J* = 15.6 Hz), 4.90 (d, 1H, downfield H of COO*CH_2_*, *J* = 15.3 Hz), 6.77-7.29 (m, 9H, arom. H + NH); MS: *m/z* (%) = 369 (M^+^, 49), 188 (14), 161 (59), 123 (100); Anal. Calcd. For C_22_H_27_NO_4_ (369.45): C, 71.52; H, 7.37; N, 3.79%. Found: C, 71.76; H, 7.15; N, 3.56%.

*4-(2-Methylpropyl)-α-methylbenzene acetic acid (4-chlorophenylcarbamoyl)methyl ester* (**3c**): m.p. 69-71 °C (from 2:1 v/v ether-petroleum ether); yield 82%; IR *ν*_max_/cm^-1^: 3396 (NH), 2958, 2927, 2869 (CH aliph.), 1745 (C=O ester), 1704 (C=O amide), 1595, 1531 (C=C); ^1^H-NMR *δ*/ppm (CDCl_3_): 0.92 (d, 6H, 2CH_3_, *J* = 6.6 Hz), 1.58 (d, 3H, CH-*CH_3_*, *J* = 7.2 Hz), 1.84-1.89 (m, 1H, *CH*-CH_2_), 2.49 (d, 2H, CH-*CH_2_*, *J* = 7.2 Hz), 3.86 (q, 1H, *CH*-CH_3_, *J* = 7.2 Hz), 4.47 (d, 1H, upfield H of COO*CH_2_*, *J* = 15.9 Hz), 4.93 (d, 1H, downfield H of COO*CH_2_*, *J* = 15.6 Hz), 7.08-7.29 (m, 9H, arom. H + NH); MS: *m/z* (%) = 375 (M+2, 20), 373 (M^+^, 62), 247 (18), 188 (93), 161 (100), 127 (30); Anal. Calcd. For C_21_H_24_ClNO_3_ (373.86): C, 67.46; H, 6.47; N, 3.75%. Found: C, 67.70; H, 6.20; N, 3.90%.

*4-(2-Methylpropyl)-α-methylbenzene acetic acid (4-sulfamoylphenylcarbamoyl)methyl ester* (**3d**): m.p. 163-165 °C (from methanol); yield 89%; IR *ν*_max_/cm^-1^: 3388, 3303, 3206 (NH, NH_2_), 2953, 2924, 2868 (CH aliph.), 1758 (C=O ester), 1685 (C=O amide), 1595, 1539 (C=C); ^1^H-NMR *δ*/ppm (CDCl_3_): 0.91 (d, 6H, 2CH_3_, *J* = 6.6 Hz), 1.58 (d, 3H, CH-*CH_3_*, *J* = 7.2 Hz), 1.86-1.91 (m, 1H, *CH*-CH_2_), 2.49 (d, 2H, CH-*CH_2_*, *J* = 7.2 Hz), 3.88 (q, 1H, *CH*-CH_3_, *J* = 7.2 Hz), 4.52 (d, 1H, upfield H of COO*CH_2_*, *J* = 15.6 Hz), 4.90 (d, 1H, downfield H of COO*CH_2_*, *J* = 15.9 Hz), 5.04 (br.s, 1H, D_2_O exchangeable NH), 7.17-7.77 (m, 10H, arom. H + NH_2_); MS: *m/z* (%) = 418 (M^+^, 4), 247 (4), 188 (79), 161 (100); Anal. Calcd. For C_21_H_26_N_2_O_5_S (418.50): C, 60.27; H, 6.26; N, 6.70%. Found: C, 60.50; H, 6.11; N, 6.67%.

*6-Methoxy-α-methylnaphthalene-2-yl acetic acid phenylcarbamoylmethyl ester* (**5a**): m.p. 111-113 °C (from ethanol); yield 85%; IR *ν*_max_/cm^-1^: 3387 (NH), 1741 (C=O ester), 1702 (C=O amide), 1535 (C=C); ^1^H-NMR *δ*/ppm (CDCl_3_): 1.69 (d, 3H, CH_3_, *J* = 7.2 Hz), 3.96 (s, 3H, O*CH_3_*), 4.02 (q, 1H, CH, *J* = 7.2 Hz), 4.47 (d, 1H, upfield H of CH_2_, *J* = 15.3 Hz), 4.94 (d, 1H, downfield H of CH_2_, *J* = 15.9 Hz), 6.75-7.78 (m, 12H, arom. H + napth. H + NH); MS: *m/z* (%) = 363 (M^+^, 37), 212 (100), 185 (95); Anal. Calcd. For C_22_H_21_NO_4_ (363.40): C, 72.71; H, 5.83; N, 3.85%. Found: C, 72.63; H, 5.88; N, 3.76%.

*6-Methoxy-α-methylnaphthalene-2-yl acetic acid (4-methoxyphenylcarbamoyl)methyl ester* (**5b**): Crystallization from benzene; m.p. 137-139 °C; yield 88%; IR *ν*_max_/cm^-1^: 3251 (NH), 1740 (C=O ester), 1661 (C=O amide), 1556, 1508 (C=C); ^1^H-NMR *δ*/ppm (CDCl_3_): 1.69 (d, 3H, CH_3_, *J* = 7.2 Hz), 3.73 (s, 3H, arom.O*CH_3_*), 3.95 (s, 3H, napth. O*CH_3_*), 4.02(q, 1H, CH, *J* = 7.2 Hz), 4.46 (d, 1H, upfield H of CH_2_, *J* = 15.6 Hz), 4.92 (d, 1H, downfield H of CH_2_, *J* = 15.6 Hz), 6.57-7.77 (m, 11H, arom. H + napth. H + NH); MS: *m/z* (%) = 393 (M^+^, 25), 213 (13), 185 (100); Anal. Calcd. For C_23_H_23_NO_5_ (393.42): C, 70.21; H, 5.89; N, 3.56%. Found: C, 70.36; H, 6.00; N, 3.70%.

*6-Methoxy-α-methylnaphthalene-2-yl acetic acid (4-chlorophenylcarbamoyl)methyl ester* (**5c**): m.p. 132-134 °C (from methanol); yield 84%; IR *ν*_max_/cm^-1^: 3389 (NH), 1749 (C=O ester), 1700 (C=O amide), 1598, 1527 (C=C); ^1^H-NMR *δ*/ppm (CDCl_3_): 1.68 (d, 3H, CH_3_, *J* = 7.2 Hz), 3.96 (s, 3H, O*CH_3_*), 4.02 (q, 1H, CH, *J* = 7.2 Hz), 4.44 (d, 1H, upfield H of CH_2_, *J* = 15.6 Hz), 4.94 (d, 1H, downfield H of CH_2_, *J* = 15.6 Hz), 6.63-7.76 (m, 11H, arom. H + napth. H + NH); MS: *m/z* (%) = 399 (M+2, 6), 397 (M^+^, 18), 213 (23), 185 (100); Anal. Calcd. For C_22_H_20_ClNO_4_ (397.84): C, 66.41; H, 5.07; N, 3.52%. Found: C, 66.45; H, 5.04; N, 3.37%.

*6-Methoxy-α-methylnaphthalene-2-yl acetic acid (4-sulfamoylphenylcarbamoyl)methyl ester* (**5d**): m.p. 202-204 °C (from methanol); yield 89%; IR *ν*_max_/cm^-1^: 3325, 3287, 3249 (NH, NH_2_), 1736 (C=O ester), 1696 (C=O amide), 1600, 1540 (C=C); ^1^H-NMR *δ*/ppm (CDCl_3_): 1.69 (d, 3H, CH_3_, *J* = 7.2 Hz), 3.98 (s, 3H, O*CH_3_*), 4.02 (q, 1H, CH, *J* = 7.2 Hz), 4.44 (d, 1H, upfield H of CH_2_, *J* = 15.6 Hz), 5.03 (d, 1H, downfield H of CH_2_, *J* = 15.6 Hz), 6.75-7.78 (m, 13H, arom. H + napth. H + NH + NH_2_); MS: *m/z* (%) = 442 (M^+^, 34), 212 (49), 185 (100); Anal. Calcd. For C_22_H_22_N_2_O_6_S (442.48): C, 59.71; H, 5.01; N, 6.33%. Found: C, 60.02; H, 4.84; N, 6.30%.

*2-Acetylaminobenzoic acid phenylcarbamoylmethyl ester* (**7a**): m.p. 209-210 °C (from ethanol); yield 80%; IR *ν*_max_/cm^-1^: 3285 (NH), 1689, 1688 (C=O), 1598 (C=C); ^1^H-NMR *δ*/ppm (DMSO-d_6_): 2.13 (s, 3H, CH_3_), 4.97 (s, 2H, CH_2_), 7.06-8.29 (m, 9H, arom. H), 10.25 (br.s, 1H, D_2_O exchangeable NH), 10.42 (br.s, 1H, D_2_O exchangeable NH); MS: *m/z* (%) = 312 (M^+^, 26), 220 (13), 162 (57), 120 (100); Anal. Calcd. For C_17_H_16_N_2_O_4_ (312.32): C, 65.37; H, 5.16; N, 8.97%. Found: C, 65.28; H, 5.24; N, 8.77%.

*2-Acetylaminobenzoic acid (4-methoxyphenylcarbamoyl)methyl ester* (**7b**): m.p. 200-202 °C (from ethanol); yield 85%; IR *ν*_max_/cm^-1^: 3337, 3264 (NH), 1704, 1686, 1668 (C=O), 1590 (C=C); ^1^H-NMR *δ*/ppm (DMSO-d_6_): 2.13 (s, 3H, COCH_3_), 3.73 (s, 3H, OCH_3_), 4.94 (s, 2H, CH_2_), 6.89-8.29 (m, 8H, arom. H), 10.10 (br.s, 1H, D_2_O exchangeable NH), 10.43 (br.s, 1H, D_2_O exchangeable NH); MS: *m/z* (%) = 342 (M^+^, 54), 220 (6), 162 (90), 123 (100); Anal. Calcd. For C_18_H_18_N_2_O_5_ (342.34): C, 63.15; H, 5.30; N, 8.18%. Found: C, 63.23; H, 5.39; N, 8.03%.

*2-Acetylaminobenzoic acid (4-chlorophenylcarbamoyl)methyl ester* (**7c**): m.p.191-192 °C (from ethanol); yield 85%; IR *ν*_max_/cm^-1^: 3424 (NH), 1658, 1618 (C=O), 1596 (C=C); ^1^H-NMR *δ*/ppm (DMSO-d_6_): 2.13 (s, 3H, CH_3_), 4.96 (s, 2H, CH_2_), 7.20-8.27 (m, 8H, arom. H), 10.39 (br.s, 1H, D_2_O exchangeable NH), 10.41 (br.s, 1H, D_2_O exchangeable NH); MS: *m/z* (%) = 348 (M+2, 6), 346 (M^+^, 14), 220 (21), 162 (83), 120 (100); Anal. Calcd. For C_17_H_15_ Cl N_2_O_4_ (346.76): C, 58.88; H, 4.36; N, 8.08%. Found: C, 58.63; H, 4.53; N, 8.29%.

*2-Acetylaminobenzoic acid (4-sulfamoylphenylcarbamoyl)methyl ester* (**7d**): m.p. 230-231 °C (from Crystallization 5:1 v/v *N*,*N*-dimethylformamide-water); yield 89%; IR *ν*_max_/cm^-1^: 3383, 3341, 3277 (NH, NH_2_), 1709, 1687 (C=O), 1596 (C=C); ^1^H-NMR *δ*/ppm (DMSO-d_6_): 2.13 (s, 3H, CH_3_), 5.00 (s, 2H, CH_2_), 7.23-8.27 (m, 10H, arom. H + NH_2_), 10.41 (br.s, 1H, D_2_O exchangeable NH), 10.59 (br.s, 1H, D_2_O exchangeable NH); Anal. Calcd. For C_17_H_17_N_3_O_6_S (391.39): C, 52.17; H, 4.38; N, 10.74%. Found: C, 52.24; H, 4.53; N, 10.83%.

### Synthesis of 4-(2,5-Dioxopyrrolidin-1-yl)benzoic acid (4-(un)substitutedphenylcarbamoyl)methyl esters ***10a-d***

*Method A:* The same method adopted in the general procedure starting with 4-(2-carboxyethylcarboxamido)benzoic acid **8** (5 mmol), potassium hydroxide (10 mmol) and the appropriate chloroacetanildes **2a-d** (10 mmol).

*Method B:* A mixture of equimolar amounts of 4-(2,5-dioxopyrrolidin-1-yl)benzoic acid (**9**) and potassium hydroxide (5 mmol) in dry *N*,*N*-dimethylformamide (5 mL) was stirred in a boiling water bath for 15 minutes. The reaction mixture was then treated with a solution of the appropriate chloroacetanilides **2a-d** (5 mmol) in *N*,*N*-dimethylformamide (3 mL) in a boiling water bath for one hour. The reaction mixture was then cooled, poured onto ice-cooled water and the separated solid was collected, dried and crystallized from a suitable solvent affording **10a-d** as colourless crystals. 

*4-(2,5-Dioxopyrrolidin-1-yl)benzoic acid phenylcarbamoylmethyl ester* (**10a**): m.p. 171-173 °C (from ethanol); yield 73% (method A); IR *ν*_max_/cm^-1^: 3359 (NH), 1782 (C=O cyclic), 1707 (C=O ester), 1605 (C=O amide), 1551, 1514 (C=C); ^1^H-NMR *δ*/ppm (DMSO-d_6_): 2.81 (s, 4H, OC-*CH_2_*-*CH_2_*-CO), 4.95 (s, 2H, COO*CH_2_*), 7.08 (t, 1H, arom. H, *J* = 7.5 Hz), 7.32 (t, 2H, arom. H, *J* = 7.8 Hz), 7.50 (d, 2H, arom. H, *J* = 8.7 Hz), 7.59 (d, 2H, arom. H, *J* = 7.5 Hz), 8.14 (d, 2H, arom. H, *J* = 8.7 Hz), 10.20 (br.s, 1H, D_2_O exchangeable NH); MS: *m/z* (%) = 352 (M^+^, 12), 260 (33), 202 (100), 93 (22); Anal. Calcd. For C_19_H_16_N_2_O_5_ (352.34): C, 64.76; H, 4.58; N, 7.95%. Found: C, 65.02; H, 4.81; N, 7.74%.

*4-(2,5-Dioxopyrrolidin-1-yl)benzoic acid (4-methoxyphenylcarbamoyl)methyl ester* (**10b**): m.p. 205-207 °C (from methanol); yield 65 and 87% (method A and B respectively); IR *ν*_max_/cm^-1^: 3356 (NH), 1779 (C=O cyclic), 1707 (C=O ester), 1608 (C=O amide), 1554, 1514 (C=C); ^1^H-NMR *δ*/ppm (DMSO-d_6_): 2.81 (s, 4H, OC-*CH_2_*-*CH_2_*-CO), 3.72 (s, 3H, O*CH_3_*), 4.91 (s, 2H, COO*CH_2_*), 6.90 (d, 2H, arom. H, *J* = 9.3 Hz), 7.49 (d, 4H, arom. H, *J* = 8.4 Hz), 8.15 (d, 2H, arom. H, *J* = 8.7 Hz), 10.05 (br.s, 1H, D_2_O exchangeable NH); MS: *m/z* (%) = 382 (M^+^, 32), 260 (11), 202 (100), 123 (45); ^13^C-NMR *δ*/ppm (APT) (DMS-d_6_): 29.24 (pyrrolidinyl *H_2_C*-3, *H_2_C*-4), 55.87 (O*CH_3_*), 64.01 (COO*CH_2_*), 114.63 (arom. *CH-*2`, *CH*-6`), 121.60 (arom. *CH*-3, *CH*-5), 121.80 (arom. *CH*-3`, *CH*-5`), 129.37 (arom. *C*-1), 130.66 (arom. *CH*-2, *CH*-6), 132 (arom. *C*-4`), 137.83 (arom. *C*-4), 156.23 (arom. *C*-1`), 165.46 (*CO*O), 165.54 (*CO*NH), 177.19 (pyrrolidinyl *CO*-2, *CO*-5); Anal. Calcd. For C_20_H_18_N_2_O_6_ (382.36): C, 62.82; H, 4.74; N%, 7.33. Found: C, 62.53; H, 4.65; N, 7.70%.

*4-(2,5-Dioxopyrrolidin-1-yl)benzoic acid (4-chlorophenylcarbamoyl)methyl ester* (**10c**): m.p. 200-202 °C (from ethanol); yield 57% (method A); IR *ν*_max_/cm^-1^: 3353 (NH), 1780 (C=O cyclic), 1706 (C=O ester), 1605 (C=O amide), 1547, 1513 (C=C). ^1^H-NMR *δ*/ppm (DMSO-d_6_): 2.81 (s, 4H, OC-*CH_2_*-*CH_2_*-CO), 4.95 (s, 2H, COO*CH_2_*), 7.38 (d, 2H, arom. H, *J* = 9.0 Hz), 7.50 (d, 2H, arom. H, *J* = 8.4 Hz), 7.62 (d, 2H, arom. H, *J* = 9.3 Hz), 8.15 (d, 2H, arom. H, *J* = 8.4 Hz), 10.35 (br.s, 1H, D_2_O exchangeable NH); MS: *m/z* (%) = 388 (M+2, 10), 386 (M^+^, 27), 260 (58), 202 (100), 126 (13); Anal. Calcd. For C_19_H_15_ClN_2_O_5_ (386.78): C, 59.00; H, 3.91; N, 7.24%. Found: C, 59.03; H, 4.16; N, 7.46%.

*4-(2,5-Dioxopyrrolidin-1-yl)benzoic acid (4-sulfamoylphenylcarbamoyl)methyl ester* (**10d**): m.p. 233-235 °C (from glacial acetic acid); yield 78% (method A); IR *ν*_max_/cm^-1^: 3325, 3227 (NH, NH_2_), 1778 (C=O cyclic), 1719 (C=O ester), 1690 (C=O amide), 1596, 1541 (C=C). ^1^H-NMR *δ*/ppm (DMSO-d_6_): 2.82 (s, 4H, OC-*CH_2_*-*CH_2_*-CO), 4.99 (s, 2H, COO*CH_2_*), 7.25 (br.s, 2H, D_2_O exchangeable NH_2_), 7.51(dd, 2H, arom. H, *J* = 1.8, 6.6 Hz), 7.77 (d, 4H, arom. H, *J* = 9.3 Hz), 8.15 (dd, 2H, arom. H, *J* = 1.8, 6.6 Hz), 10.56 (br.s, 1H, D_2_O exchangeable NH); MS: *m/z* (%) = 431 (M^+^, 16), 260 (24), 202 (100), 172 (18); Anal. Calcd. For C_19_H_17_N_3_O_7_S (431.41): C, 52.89; H, 3.97; N, 9.74%. Found: C, 52.56; H, 4.24; N, 9.63%.

### Anti-inflammatory Activity Screening

Anti-inflammatory activity screening for the prepared compounds was determined *in vivo* by the standard acute carrageenan-induced paw oedema method in rats [21,22,23]. Wister albino rats of either sex (pregnant female animals were excluded) weighing 160-190 g were divided into 19 groups of six animals each. Ibuprofen and naproxen (reference standards) and the tested compounds (**3a-d**, **5a-d**, **7a-d** and **10a-d**) dissolved in DMSO, at a dose of 50 mg/kg body weight, were administered intraperitoneally, while the control group received DMSO, 1 hour before induction of inflammation. Carrageenan paw oedema was induced by subcutaneous injection of 1% solution of carrageenan in saline (0.1 mL/rat) into the right hind paw of rats. Paw volumes were measured volumetrically after 4 hours with a 7140 plethysmometer (Ugo Basile, Italy) and compared with the initial hind paw volume of each rat for determining the oedema volume. Data were collected, checked and revised. Quantitative variables from normal distribution were expressed as means ± SE "standard error". The significant difference between groups was tested by using one-way ANOVA followed by Tukey HSD test at p < 0.05. The anti-inflammatory activity was expressed as percentage inhibition of oedema volume in treated animals in comparison with the control group (Table 1):

$$\%\text{ Inhibition of oedem }=\frac{Vc-Vt}{Vc}x100$$

where, *Vc* and *Vt* are the volumes of oedema for the control and drug-treated animal groups.

### Ulcerogenic liability

The ulcerogenic liability was determined in albino rats following the previously reported standard method [23,24]. Rats of either sex (pregnant female rats were excluded) weighing 120-140 g were divided into nine groups of five animals each. The animals were fasted 18 hours before drug administration. Ibuprofen and naproxen (reference standards) and the tested compounds at a dose 50 mg/kg body weight, were suspended in saline solution by the aid of few drops of Tween^®^ 80 and were administered orally for three successive days to fasted rats. The control group animals were given saline with few drops of Tween^®^ 80. One hour after the last dose, the animals were sacrificed by cervical dislocation and the stomach was removed, opened along the greater curvature and rinsed with saline. The gastric mucosa was examined with a magnifying lens (10 x) for the presence of lesions and erosions. The ulcer index was calculated (Table 2) and the degree of ulcerogenic effect was expressed in terms of:

1. Percentage incidence of ulcer divided by 10.
2. Average number of ulcers per stomach.
3. Average severity of ulcers.

The ulcer index is the value that resulted from the sum of the above three values. 

### Measurement of PGE_2_ Level

Measurement of PGE_2_ level was determined by the previously described six-day-old air pouch standard method in rats [25,26]. Male albino rats weighing 200-250 g were divided into six groups of six animals each. The air pouch was induced as follows: on the first day of the experiment, 20 mL of air was injected subcutaneously in the back of each rat. Two days later, another 10 mL of air was injected at the same site. On the fifth day after the first injection, a further 10 mL of air was injected into the pouch. Then, 24 h later and before injecting the pouch with carrageenan (2 mL of 1% solution in saline), four groups of animals were treated orally with the tested compounds (**3c, 5a, 7a** and **10b**) at a dose of 50 mg/kg body weight, one group with indomethacin (reference standard) at a dose of 10 mg/kg body weight suspended in saline solution by the aid of few drops of Tween^®^ 80 and the last group with sterile saline (control group). All injections were conducted under light ether anaesthesia. Then 6 h after the carrageenan injection, animals were lightly anaesthetised with ether and the contents of the pouch were aspirated using a Pasteur pipette and transferred into graduated plastic tubes kept in ice. The bulk of the exudates was frozen and stored at -20 °C until required for PGE_2_ assay. PGE_2_ was measured by an ELISA (Beckman Biomek^TM^ 1000 Automated Laboratory Workstation apparatus) technique using PGE_2_ assay kit supplied by R&D Systems (Minneapolis, USA & Canada) according to the manufacturer’s specifications. Data were collected, checked and revised. Quantitative variables from normal distribution were expressed as means ± SE "standard error". The significant difference between groups was tested by using one-way ANOVA followed by Dunnett`s test at p < 0.05 (Table 3).
